# Weather influences *M*. *arvalis* reproduction but not population dynamics in a 17-year time series

**DOI:** 10.1038/s41598-019-50438-z

**Published:** 2019-09-26

**Authors:** Patrick Giraudoux, Petra Villette, Jean-Pierre Quéré, Jean-Pierre Damange, Pierre Delattre

**Affiliations:** 1 Chrono-environnement, Université de Bourgogne Franche-Comté/CNRS usc INRA, F-25030 Besançon, Cedex France; 2 CBGP, INRA, Campus international du Baillarguet, Montferrier/lez, France

**Keywords:** Population dynamics, Population dynamics

## Abstract

Rodent outbreaks have plagued European agriculture for centuries, but continue to elude comprehensive explanation. Modelling and empirical work in some cyclic rodent systems suggests that changes in reproductive parameters are partly responsible for observed population dynamics. Using a 17-year time series of *Microtus arvalis* population abundance and demographic data, we explored the relationship between meteorological conditions (temperature and rainfall), female reproductive activity, and population growth rates in a non-cyclic population of this grassland vole species. We found strong but complex relationships between female reproduction and climate variables, with spring female reproduction depressed after cold winters. Population growth rates were, however, uncorrelated with either weather conditions (current and up to three months prior) or with female reproduction (number of foetuses per female and/or proportion of females reproductively active in the population). These results, coupled with age-structure data, suggest that mortality, via predation, disease, or a combination of the two, are responsible for the large multi-annual but non-cyclic population dynamics observed in this population of the common vole.

## Introduction

Multiannual fluctuations in temperate small mammal populations have eluded consistent explanation for more than 100 years, and continue to do so^1^. These fluctuations are of interest because many of the species that exhibit them have a large influence on the community dynamics of their respective ecosystems: some are perceived as pests for crops, grasslands, and tree seedlings, and are targeted for control, but are also considered keystone species in their native range^2^. Reaching high population densities, they are at the base of temperate and arctic food webs, maintaining large and rich communities of predators, and modifying nutrient cycling, soil aeration, and microorganism communities^3–6^.

The common vole, *Microtus arvalis*, is one such species that has received much attention from both researchers and rodent control organizations, and is considered a serious agricultural pest and human health hazard in much of its range^7–14^. Common vole populations thrive in managed grasslands, where fodder production can range from 5–12 tonnes of dry matter/ha/year^15^. Common voles can also concentrate in marginal habitats such as grassy field borders and leguminous semi-permanent fields (alfalfa, clover, etc.), recolonizing adjacent intensively-farmed croplands during the growing season when suitable food and protective cover become available and intensive plowing has ceased^16^.

Common vole population dynamics appear to be strongly correlated with landscape configuration; in comparing time series of vole populations in various regions of France, Delattre *et al*.^17^ reported a variety of patterns, ranging from low-density populations prone to local extinction in homogeneous intensively farmed agricultural landscapes, to multi-annual large-amplitude fluctuations in pastoral landscapes consisting of permanent grassland (see also^18–20^). In Spain, common vole range expansion and the development of regular outbreaks causing considerable crop damage in the Castilla-y-León region appears to have been driven largely by landscape changes, specifically an increase in irrigated crops and alfalfa cultivation^7,21^. Conversely, in France, agricultural changes in western wetlands have resulted in almost 50% of the pastures being converted to drained agricultural production, with a subsequent decrease in the frequency and intensity of vole population peaks^22^.

It is clear from the above examples that landscape composition is important for vole population dynamics, but which demographic parameters (e.g. reproduction and/or mortality and/or dispersion) respond to landscape is less clear. One proposed mechanism by which landscape may act on vole population dynamics is via predator-induced mortality; Delattre *et al*.^17^ suggested that landscape configuration could enhance or reduce the dominance of specialist predators within the predator community, with cycles more likely when destabilizing specialists are dominant. This has been further expanded into the conceptual Trophic Ratio of Optimal to Marginal Area Integrated Model (TRIM)^23^, in which productivity and landscape configuration drive source-sink dynamics in vole populations and the relative importance of specialist and generalist predators, which together can interact to generate the range of population dynamics observed in rodent populations. In Fennoscandia, it has been suggested that snowpack influences the effects of specialist and generalist predators, with snowpack serving to isolate specialist predators and the voles from the stabilizing influences of generalist predators^24^. However, Mougeot *et al*.^25^ suggest that weasels might follow rather than cause the vole cycles in north-western Spain.

Conversely, much of the modelling work on cyclic population dynamics has suggested that reproductive parameters (namely, age at maturity and length of reproductive season) are the drivers of changes in population growth rates^26,27^. In mountain water voles (*Arvicola terrestris*), another cycling grassland species, impaired reproduction as the result of stress has been suggested as a mechanism for the decline phase of the cycle^28^. This has been somewhat corroborated by Cerqueira *et al*.^29^, who reported population senescence during population declines, and a re-analysis of the reproduction data presented by these authors shows a significantly lower proportion of reproductive females at the end of the reproductive season during the decline phase compared to the other phases, adding further weight to the idea that reproduction is the demographic parameter driving the decline phase of the cycle in this species. Regarding the common vole, in a one year survey carried out in western France, Pinot *et al*.^30^ suggested that winter declines could reflect negative effects of density dependence on reproduction rather than changes in mortality rates.

Cornulier *et al*.^31^ suggested a common climatic driver to cycle amplitude dampening associated with a reduction in winter population growth throughout Europe, attributed to increasingly mild winters, in 10 populations of’grass eating’ voles out of the 12 included in the study. The role of meteorological conditions on both common vole reproduction and population dynamics was reported some time ago in the polders of Vendée, France^32,33^, and is still considered an important cause of multi-annual population fluctuations, at least in agricultural landscapes of central Europe^34–36^. The general idea is that cold winters and late springs delay reproduction, limiting the increase of vole populations during a subsequently shortened reproduction season; however, most of the studies undertaken, including Delattre and colleagues’s, investigated the patterns of population dynamics from historical data, with no demographic data allowing for the isolation of specific parameters such as reproduction, mortality, etc. that might explain the observed patterns.

In this paper we consider a 17 year (1979–1996) time series of *M*. *arvalis* population fluctuations in eastern France, in a landscape of permanent grassland where the ratio of optimal habitat is at its maximum in farmland, and grassland productivity averages 5.3 tons of hay (dry matter, DM).ha^−1^.an^−1^ ^37^. We explored the relationship between meteorological conditions (temperature and rainfall), female reproductive activity, population growth rates and potential cyclicity in a population of this grassland vole species.

## Material and Methods

### Study area

Data were collected from August 1979 to October 1996 at Septfontaines – Le Souillot (6.18°E, 46.97°N), in an area of 1400 ha (800 ha of farmland, 600 ha of forest), at an average altitude of 750–800 m above sea level^38^. There, 100% of the farmland was permanent grassland used for pasture and (high grass) meadow for cattle feeding in winter (minimum 6 months, November–March), with a productivity ranging from 2.3 tonnes of dry matter.ha^−1^.an^−1^ for areas of lower productivity and up to 9.0 tonnes of dry matter.ha^−1^.an^−1^ for rich meadows^37^. Legumes (alfalfa, clover) and silage were (and still are) not allowed with respect to the European Protected Geographical Indication specifications of the locally produced “Comté” cheese. A KML file with the bounding box of the study area is provided in the Supplementary Material.

### Trapping and dissection

Rodents were captured using INRA traplines. INRA live traps (15 × 5 × 5 cm) are suitable for species of body mass less than 50 g. Each line consisted of 34 live traps spaced 3 meters apart. Distances between traps corresponds to a standard design^39–41^ for which a minimum of 2 traps should (theoretically) be available in each *M*. *arvalis* home range. Trap lines were set up for three nights and checked every day. Traplines were all set in grasslands but their position was changed from one session to the other in order to avoid over-trapping. A minimum distance of 100 m was kept between two traplines.

Animals were euthanized by cervical dislocation, weighed and dissected for sex, reproductive status and age determination. Relative age was estimated by weighing the crystalline eye lenses after drying^42,43^. Pregnant or lactating females, as well as those with visible *corpus luteum*, were considered reproductive.

Trapping and animal handling was carried out in full accordance with the relevant European guidelines (Directive 86/609/EEC) and national regulations. The rodent species investigated in this study does not have protected status (see IUCN and CITES lists), and is listed as a pest, subject to control, under *Article L201-1* of the *Code Rural et de la Pêche Maritime* of French law. INRA (*Institut National de la Recherche Agronomique*), the umbrella organization under which the field work was carried out, created its first ethical committee in 1998, 2 years after the end of our study. It was therefore impossible to get formal ethical approval prior to the study, and INRA does not issue retroactive ethical approvals. A similar research protocol used from 2014 to 2017^44^ received full approval from the *Comité d*’*Ethique Bisontin en Expérimentation Animale* (CEBEA No. 58).

### Meteorological data

Meteorogical data were kindly provided by Meteo-France. Monthly temperatures from 1983 to 1996 were from the Levier station (altitude 713 m), 6 km from the study area. From 1979 to 1982, temperature data from this station were not available, thus we estimated monthly values in Levier based on temperatures recorded at the Pontarlier station (altitude 831 m, 20 km from Levier and 15 km from the study area), after calibrating the two stations over the period 1983–1986 with linear regression (adjusted R^2^ = 0.99, p < 0.00001). Assuming that low temperature was a limiting factor with possibly delayed effects, and following Krebs’s opinion^1^ that “the time scale of rodent dynamics is monthly or even weekly” (due to population turnover), we chose to investigate the effect of the deviation from the monthly average minimum temperature of the current month and of the previous three months before a focal event on reproduction parameters and population dynamics.

Monthly rainfall data were from the Levier station from 1979 to 1996. The variable under study was the deviation from the average rainfall of the month during the study period. As for temperature, hypothesizing delayed effects, we considered rainfall of the current month and of the previous three months of a focal event.

### Statistical analyses

We investigated density dependence after log-transforming population estimates^1,45,46^ obtained from the mean of the whole sampling region. In brief, we used relative density estimates from autumn and from spring separately, calculated autocorrelation coefficients and fitted autoregressive models of order 1, 2 and 3 to the data.

$${R}_{t}={a}_{0}+{a}_{1}{X}_{t-1}+{a}_{2}{X}_{t-2}+{a}_{3}{X}_{t-3}+{\varepsilon }_{t}$$

where

$${R}_{t}={X}_{t}-{X}_{t-1}=\,\mathrm{ln}\,({N}_{t}/{N}_{t-1})$$

$${X}_{t}=\,\mathrm{ln}({N}_{t})$$

*N*_*t*_ = abundance estimate for year *t*, and

*t* = time in year

*ε*_*t*_ = the regression residuals.

Other data were either counts (e.g. a number of individuals) or presence/absence (e.g. reproductive/not reproductive) and were modelled against independent variables (e.g. relative age, temperature at given months, rainfall, etc.) using Generalized Linear Models (GLM) with Poisson log or Binomial logit link functions, respectively^47^ since 1982 (all estimates before 1982 but one - May 1981 - are just indicative, since they are based on very small sample sizes).

Delayed effects of temperature and rainfall were investigated by modelling the response variable (either the reproductive status or the population growth rate) against meteorological variables of the current and 3 previous months using GLM (Binomial or Gaussian link function, according to case), as

$${x}_{t}={a}_{0}+{a}_{1}\,{y}_{t}+{a}_{2}\,{y}_{t-1}+{a}_{3}\,{y}_{t-2}+{a}_{4}\,{y}_{t-4}+{\varepsilon }_{t}$$

where

*x*_*t*_ = the response variable (reproductive status or population growth rate)

*y*_*t*_ = the deviation from the average at time *t* (minimum temperature of the month or rainfall)

*t* = time in month

*ε*_*t*_ = the regression residuals

All statistical analyses were performed in R (version 3.4.1)^48^.

## Results

Over the 17 years of our study, we set up 1188 traplines and captured 8504 common voles (Table 1). Sample sizes ranged from as low as 1 animal per trapping session (July 1980 and August 1981) to as high as 72 animals (April 1988).

**Table 1:** Number of traplines and specimens caught. time, “7908” reads August 1979, etc.; n, the number of traplines; NS, animals not sexed.

Time	n	Male	Female	NS	Total	Time	n	Male	Female	NS	Total
7908	4	26	13	0	39	8710	33	130	128	0	258
7911	3	0	0	111	111	8804	72	208	116	0	324
8007	1	0	0	26	26	8807	21	146	122	0	268
8010	3	0	0	122	122	8810	33	300	292	1	593
8105	10	1	0	0	1	8904	54	125	77	0	202
8108	1	0	0	0	0	8907	23	55	24	1	80
8204	17	3	3	1	7	8910	35	49	41	1	91
8207	14	8	10	0	18	9004	70	46	32	0	78
8210	12	31	26	0	57	9007	23	7	3	0	10
8304	12	28	24	0	52	9010	32	63	62	0	125
8308	17	90	101	1	192	9104	28	47	20	0	67
8310	13	84	100	0	184	9107	23	143	134	0	277
8404	16	58	30	0	88	9110	25	182	221	0	403
8407	16	54	48	0	102	9204	27	13	10	0	23
8409	18	268	269	0	537	9207	22	49	42	0	91
8504	16	280	121	0	401	9210	28	137	154	0	291
8506	15	168	128	0	296	9304	46	81	44	0	125
8508	26	277	308	0	585	9307	22	99	83	0	182
8510	15	205	191	0	396	9310	28	137	138	0	275
8604	28	157	88	0	245	9404	24	23	17	0	40
8606	15	39	21	0	60	9410	24	8	2	0	10
8608	23	111	92	0	203	9504	23	3	1	0	4
8610	23	201	148	0	349	9510	24	25	7	0	32
8704	33	104	50	0	154	9604	24	9	8	0	17
8706	30	39	24	0	63	9610	24	73	98	0	171
8708	19	107	72	0	179	total	1188	4497	3743	264	8504

### Population dynamics patterns

**Figure 1:**
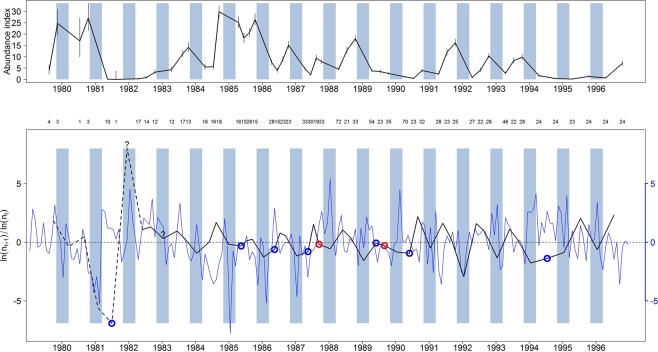
Abundance index variations, growth ratio and deviation from the monthly average temperature. Upper panel: blue box, winter period (November 1 to March 15); vertical bars, 95% confidence interval (Poisson). Lower panel: numbers above, number of traplines; growth rateas *log*(*N*_*t*+1_) − *log*(*N*_*t*_). Estimates before 1982 are just indicative since grounded on very small sample size. Circles, population decrease out of the winter season; blue, spring decrease, red, summer decrease (in 1994 there was no summer sampling, subsequently decrease was observed from spring to autumn with no mid-way estimate), ? winter increase. the blue line is the deviation from the 1979–1996 average temperature of the month. Rightscale of the vertical axis (in blue) corresponds to temperature deviations (in °C).

Population dynamics showed a classical pattern with characteristic seasonal population declines almost every winter due to concomitant reproduction stoppages in combination with winter mortality, with the exception of winter 1982–1983 and perhaps winter 1981–1982 in which populations didn’t decline over winter (Fig. 1, upper panel). Inter-annual variations of large amplitude were also observed. Figure 1, lower panel, shows log transformed population growth rate; we observed 7 spring and 2 summer population declines during the reproductive period. In detail:

- 14 winters show clear decreases in population abundance, 2 show increases or stability; the first of these 2 latter paradoxical exceptions corresponds to an imprecise estimate of population change in 1981–1982, from close to zero in the autumn to 0.3 individuals.trapline^−1^ in the next spring; this is geometrically exaggerated on the chart by the log scale. The second in 1982–1983 corresponds to more reliable estimates and provides clear evidence of a lack of population decline in winter (stability or even a slight increase).
- At least 6 winter declines are followed by a spring decline (7 if 1981 is included).
- At least two summer declines could be observed:

- One (1987) in late summer after a winter and spring decline and early summer increase.
- One (1989) following a winter and spring decline.

- One (1994) spring-to-autumn decline (no summer estimate available) could also be observed.

Except for winter declines, attributable to seasonal reproductive patterns and subsequent uncompensated mortality, we did not observed temporal regularity.

### Density dependence

We did not detect delayed density dependence, and found evidence of direct density dependence in autumn only (Table 2): the larger the population growth at year *t*, the more likely a decline between year *t* and *t* + 1.

**Table 2:** Parameters of the autoregressive model (Autumn relative densities), Adjusted R^2^ = 0.49.

	Estimate	Std. Error	t value	Pr (>|t|)
(Intercept)	2.2978	0.7579	3.03	0.0114
*X*_*t*−1_	−0.7542	0.1995	−3.78	0.0030
*X*_*t*−2_	−0.1669	0.1901	−0.88	0.3985
*X*_*t*−3_	−0.2089	0.2196	−0.95	0.3619

We also detected positive correlations between autumn and early spring densities (adjusted R^2^ = 0.70, p < 0.0001) and between spring and autumn densities (adjusted R^2^ = 0.34, p = 0.01); this might however be a trivial result, quantifying something that can be observed visually in Fig. 1: the time series considered is a sequence of periods of several successive years where densities are on average high, and of periods where densities are on average low, whatever the season.

### Age structure and reproduction parameters

**Figure 2:**
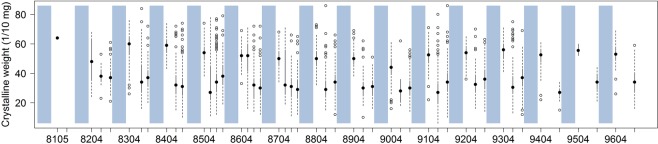
Age structure variations. Black circles, median; vertical bars, whisker box (solid line, inter-quartile interval; dotted line, whisker range, maximum 1.5x the interquartile interval; empty circles, outliers.

The age structure of the population varied in accordance with seasonal reproduction; lack of breeding over winter led to population aging until early spring when reproduction started again, and breeding throughout the summer until at least October. This led to rejuvenated populations from spring to summer, and sometimes into autumn (Fig. 2).

**Figure 3:**
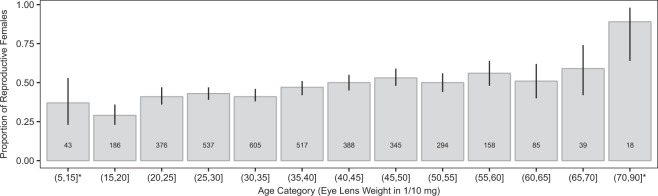
Proportion of reproductive females in each age category (defined by cristalline lens weight in 1/10 mg). Error bars are 95% confidence intervals, numbers within bins are sample sizes.

The proportion of reproductive females increased with age (Fig. 3 and Table 3), from a baseline of 37% in the younger female age class (Fig. 3). Notably, the minimum crystalline lens weight for a pregnant female in our sample was 1.1 mg, which corresponds to an animal of about 13 days old^42,49^. Its body mass was 15 g (3/4 of the average body mass of an animal fully grown) and it carried 5 foetuses. Moreover, we found that the proportion of reproductive females increased with age but that they produced fewer foetuses (Table 3); however, the effect size could be considered nominal since models accounted for an extremely small proportion of the total deviance (R^2^ = 0.01 for the two models) with an extremely low proportion of old females in the total population (Fig. 3).

**Table 3:** Parameters of GLM: reproductive status (1/0) (Binomial logit link function) and number of foetuses of reproductive females (Poisson log link function) against relative age (expressed as crystalline lens weight, cryst).

		Estimate	Std. Error	z value	Pr (>|z|)
Reproductive status	(Intercept)	−0.9060	0.1102	−8.22	0.0000
	cryst	0.0196	0.0028	7.04	0.0000
Number of foetuses	(Intercept)	1.7519	0.0443	39.56	0.0000
	cryst	−0.0032	0.0011	−2.89	0.0038

### Reproduction parameters and declines

**Figure 4:**
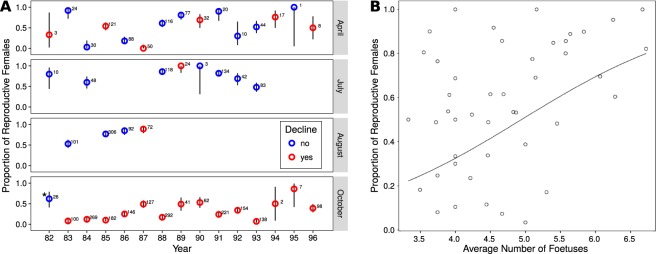
(**A**) Proportion of reproductive females in April and October from 1982 to 1996 (and in July and August when available.) Population dynamics are indicated with color - populations were either declining (red) or stable/increasing (blue). (**B**) Relationship between the number of foetuses per female and the proportion of reproductive females in the population. The solid line represents the fit of a Binomial GLM (R^2^ = 0.22, p < 0.0001).

The proportion of reproductive females was highly variable between years at the beginning of the reproductive season (April). Declines did not correlate with variations in the ratio of reproductive females; declines could (and most often did) occur with proportions of reproductive females similar to and sometimes higher than those observed during population growth (Fig. 4A). We observed similar patterns for the other months (July and August, Fig. 4A).

In October, close to the end of the reproductive season, large variations in the proportion of reproductive females could be observed between years (Fig. 4A). Moreover, the average number of foetuses per female and the proportion of reproductive females in the population were positively correlated (Fig. 4B).

Furthermore, we did not detect correlations between the population growth rate and the ratio of reproductive females and/or the number of foetuses, alone or combined, including interactions, at the beginning of the time span on which the growth rate was computed (e.g. between the reproduction parameters of April and the growth rate computed from April to July, etc.). We also took the sum of the proportion of reproductive females in April and October as a proxy of the reproduction season duration, with the assumption that the larger the ratio in April, the earlier the reproduction began that year, and the larger the ratio in October, the longer reproduction continued into the autumn. Here again we did not detect any correlation between the population growth rate from April to October and this proxy.

### Meteorological conditions and reproduction

The ratio of reproductive females in April was significantly lower when previous months were colder than the average (Table 4). For the other periods and meteorological variables, correlations between the ratio of reproductive females, temperature and rainfall, although statistically highly significant, appeared complex, often time-lagged, and hard to interpret unequivocally (Tables 4 and 5). Clearly, meteorological conditions have an impact on reproduction, but differ among circumstances (e.g. rainfall as well as temperature may impact reproduction positively or negatively from one month to the other).

**Table 4:** Parameters of GLM (Binomial logit link function): reproductive status (1/0) against the deviation from the average minimum temperature of the month and each of the previous months.

		Estimate	Std. Error	z value	Pr (>|z)
April R^2^ = 0.4	(Intercept)	0.4624	0.2655	1.74	0.0816
	T04	0.4425	0.0929	4.76	0.0000
	T03	0.3910	0.0669	5.84	0.0000
	T02	0.0904	0.0381	2.38	0.0175
July R^2^ = 0.7	(Intercept)	−0.3024	0.8691	−0.35	0.7279
	T07	0.6427	0.1154	5.57	0.0000
	T06	−0.6470	0.1159	−5.58	0.0000
August R^2^ = 0.97	(Intercept)	5.5039	1.1180	4.92	0.0000
	T08	0.0448	0.1479	0.30	0.7620
	T07	−0.4183	0.0836	−5.00	0.0000
October R^2^ = 0.4	(Intercept)	0.4624	0.2655	1.74	0.0816
	T10	0.4425	0.0929	4.76	0.0000
	T09	0.3910	0.0669	5.84	0.0000
	T08	0.0904	0.0381	2.38	0.0175

“T04” reads deviation from the average minimum temperature of April of the study period, etc. Last terms have been dropped when statistically not significant.

**Table 5:** Parameters of GLM (Binomial logit link function): reproductive status (1/0) against the deviation from the average rainfall of the month and each of the previous months.

		Estimate	Std. Error	z value	Pr (>|z|)
April R^2^ = 0.2	(Intercept)	−0.9737	0.3857	−2.52	0.0116
	R04	0.0057	0.0013	4.41	0.0000
	R03	0.0057	0.0015	3.90	0.0001
	R02	0.0015	0.0018	0.84	0.4030
	R01	−0.0050	0.0013	−3.77	0.0002
July R^2^ = 0.56	(Intercept)	5.9047	0.9299	6.35	0.0000
	R07	0.0010	0.0061	0.17	0.8649
	R06	−0.0145	0.0039	−3.71	0.0002
	R05	−0.0176	0.0043	−4.05	0.0001
	R04	−0.0068	0.0031	−2.18	0.0292
October R^2^ = 0.2	(Intercept)	−0.9737	0.3857	−2.52	0.0116
	R10	0.0057	0.0013	4.41	0.0000
	R09	0.0057	0.0015	3.90	0.0001
	R08	0.0015	0.0018	0.84	0.4030
	R07	−0.0050	0.0013	−3.77	0.0002

“R04” reads deviation from the average rainfall of April of the study period, etc. Last terms have been dropped when statistically not significant. We did to detect regression coefficients significantly different from zero in August (data not shown).

### Meteorological conditions and population dynamics

We did not detect any effect, delayed or direct, of temperature or rainfall, alone or combined (data not shown), on population growth (Table 6). For example, Fig. 1 shows that winters 1988–1989, 1989–1990, and 1993–1994 were among the warmest, but voles still underwent the “classical” winter declines, which in 1989 and 1994 were even followed by spring declines. Winters from 1984 to 1986 were among the coldest but this was the period when vole densities were higher, though there were some spring declines that were largely compensated by further population growth. 1989–1991 and 1994-early 1996 were periods when vole densities were lower, but these periods were far from the coldest; and while spring and summer 1984 were among the coldest, we observed one of the most dramatic increases in abundance in the entire time series. In short, population growth rates, whether they be positive or negative, appear wholly uncorrelated with meteorological conditions, or at least temperature and rainfall.

**Table 6:** Parameters of GLM (Gaussian link function): population growth (*ln*(*N*_*t*_/*N*_*t*−1_)) against the deviation from the average minimum temperature of the month and each of the previous months, and against the deviation from the average rainfall of the month and each of the previous months.

		Estimate	Std. Error	t value	Pr (>|t|)
Temperature	(Intercept)	−0.1408	0.7743	−0.18	0.8573
	T0	0.0534	0.0956	0.56	0.5817
	T1	0.0985	0.0835	1.18	0.2509
	T2	−0.0695	0.0951	−0.73	0.4728
	T3	−0.1012	0.0647	−1.56	0.1320
Rainfall	(Intercept)	−0.4369	0.6526	−0.67	0.5102
	R0	0.0007	0.0036	0.20	0.8437
	R1	0.0026	0.0028	0.91	0.3709
	R2	0.0024	0.0027	0.89	0.3848
	R3	0.0012	0.0024	0.50	0.6205

“T0” and “R0” read deviation from the average minimum temperature of April and from the average rainfall at *t*_0_, “T1” and “R1” at *t*_−1_ (in month), etc.

## Discussion

Using a 17-year time series of *Microtus arvalis* abundance and reproductive activity and concurrent weather data, we have explored the relationship between meteorological conditions and reproduction in this species, as well as reproduction and population growth rates. We have detected significant but complex relationships between meteorological conditions (temperature and rainfall) and female reproduction, but we did not detect any clear relationship between female reproduction and population growth rates.

Spitz^32,33^ was the first to propose a meteorological model predicting the timing of yearly population peaks of *M*. *arvalis*, basing their model on winter temperatures in polders of western France where large-amplitude variations were primarily seasonal. Several other studies in Europe have correlated meteorological conditions to *M*. *arvalis* population dynamics: Cornulier *et al*.^31^ have demonstrated consistent dampening of cycle amplitude associated with a reduction in winter population growth on a continental scale, suggesting a common climatic driver; Imholt *et al*.^34^, using linear methods, reported that weather parameters in winter and early spring are related to regional outbreak risk in eastern Germany; and Esther *et al*.^35^, using regression trees, found that between 12 and 20 weather parameters, depending on population and location, could influence relative vole density. In this last study, the number of days with snow cover in December and March, rainfall in spring, and the maximum temperature in October were all identified as key predictors of spring population densities the following year, and monthly maximum temperatures between February and June and the amount of precipitation in April and July were found to be correlated with population densities in fall. In short, meteorological conditions are generally considered to be important in *M*. *arvalis* populations dynamics.

Somewhat contrary to the studies listed above, we did not detect any impact of weather variation, direct or delayed, on population dynamics. However, we found strong direct and delayed correlations between weather parameters and reproduction: low temperature in winter delayed reproduction, decreasing the proportion of reproductive females the following spring. While we found other parameters and their combinations to have an impact on reproduction, these results appear complex and are quite hard to interpret in terms of the underlying ecological processes. We also acknowledge that the list of weather variables we considered was not exhaustive, and that linear models might not always capture the observed complexity. Pucek *et al*.^50^ suggested that ambient temperature may play a role in Polish bank vole (*Myodes glareolus*) population dynamics via it’s effect on plant growth and subsequent food availability, and Tkadlec *et al*.^36^ found that between North Atlantic Oscillation (NAO) index data and crop yield indices, the latter were better predictors of population change in voles in the Czech Republic. Blank *et al*.^51^ found that topography and soil properties affect the local risk of common vole outbreaks in Eastern Germany. In the present study, we were unable to include plant productivity as a predictor because reliable data (NDVI, etc.) were not available at the relevant temporal and spatial scales and resolution over the study period; however, this may be a mechanism by which temperature and rainfall can influence reproduction in this population of *M*. *arvalis*, and deserves further study.

We found that population dynamics were completely disconnected from variation in reproduction (and thus also from variation in weather conditions). We did not find correlations, direct or delayed, between female reproduction parameters and population growth rate. Strikingly, apart from regular annual winter population decreases, we even observed population declines in spring and summer in periods when reproduction, as measured by the proportion of reproductive females and the number of foetuses, was among the most intense. Here it does not appear that reproductive change is an important component of population density change in our system.

This is in contrast to some of the recent work on *M*. *arvalis* populations elsewhere; Pinot *et al*.^30^ reported on a *M*. *arvalis* population in which steeper over-winter declines were attributed to more pronounced reductions in winter reproduction and recruitment following higher October densities; density appeared to have a slightly positive effect on survival, and it was concluded that mortality did not drive the steeper declines observed at high density. This is consistent with one of the competing paradigms regarding cyclic rodent populations: population cycling is a result of changes in reproductive parameters and not mortality. Modeling work has suggested that phase-specific changes in female age or size at sexual maturity, aided by changes in juvenile survival, are sufficient to generate multi-annual population cycles in rodents^26,27^, and empirical work in a variety of systems and rodent species has suggested that the length of the breeding season also plays a role (^52,53^, reviewed in^1^). If changes in reproduction are not responsible for the changes in population growth rates, then mortality must be considered. The analysis of the relative age structure of our population of *M*. *arvalis* each year conforms to seasonal reproductive patterns and does not indicate, for example, a lack of recruitment of specific age categories (e.g. young individuals during the reproductive season). This would have been the case if differential mortality occurred according to age categories (e.g. infanticide, differential predation or diseases, etc.). This rules out, for example, specific nest mortality as a unique parameter of the decline. Weather being excluded, social interactions, predation and diseases, alone or combined remain as alternative sources of mortality. Since the 1990s, most population ecologists have rejected intrinsic or self-regulation hypotheses as a possible explanation for population fluctuations^1^, thus predation and/or diseases become the most likely factors driving population dynamics here. High population densities may create conditions favourable to the spread of parasites and diseases, both through increased transmission but also increased host susceptibility^54–56^, and also to predator concentration in the area.

We did not detect delayed density dependence whatever the season considered; however, large but non-cyclic variations in density, with high density peaks >10 individuals.trapline^−1^ of 2–6 years, were observed over the 17 year of our study. Based on Spitz’s^41^ calibrations, a peak of 10 individuals.trapline^−1^ should correspond to approximatively 100 individuals.ha^−1^ on a landscape scale (5–25 km^2^). Peaks of up to 20–30 individuals.trapline^−1^ on average were observed in our study, which corresponds to 200–300 individuals.ha^−1^ with large variability between plots (1–5 ha) and peaks of a magnitude of 1000 to 2000 individuals.ha^−1^ locally. This is corroborated by 2–3 km of transects walked across grassland which noted the presence of indices (holes, corridors, feces) in every 10 m interval (see e.g.^18,19^).

This pattern, of large but non-cyclic variations in density, is consistent with the range of dynamics observed in *M*. *arvalis*. Mackinrogalska *et al*.^57^, in an extensive review (36 data sets), found 6 populations with cycles shorter than 3 years, 26 ranging between 3 and 4.9 years, 6 larger than 5 years and 4 with no evidence of cyclicity in various populations of Europe. More recent work has demonstrated a similarly high degree of variability: using autoregressive models, Lambin *et al*.^24^ provided evidence that patterns observed in common vole population fluctuations in western France from the 1970s until the early 2000s show 3 year cycles; Luque-Larena *et al*.^7^ found a 5 year cycle from the mid 1980s to 2010 in northern Spain; Tkadlec *et al*.^36^ did not detect delayed density dependence in 71 districts of the Czech Republic in a set of 1968–1988 time series; and Gouveia *et al*.^58^ identified regional synchronies decreasing with distance in the same areas between 2000–2014.

Though Mackinrogalska’s review^57^ used the s-index to determine cyclicity^59,60^, a measure of cyclicity which has been criticized elsewhere^1^, it and the other studies listed above clearly show the considerable spatial and temporal variation in demographic behaviour within the same species^23^. They also show that simple unified explanations for all of those patterns have yet to be found. As discussed in Lambin *et al*.^24^, comparisons between the amplitude of population density variations in *M*. *arvalis* are quite difficult because most studies are grounded on various index-based trapping methods or qualitative historical records, and where density estimates exist they are scale-dependent. Furthermore, landscape descriptions generally do not provide quantified information on habitat composition and structure, and sampling design descriptions are rarely complete enough to ensure full comparability between studies. To our knowledge, single-factor hypotheses have considerably increased our understanding of possible causes for vole population regulation, but did not provide comprehensive explanations regarding the diversity of vole population dynamics (see^1^ for a review); this is demonstrated by the fact that none of those single-factor frameworks have provided practical and efficient ways to control vole pests. On the contrary, vole control methods based on multifactorial hypotheses, that take specificities of the local environment into account, have proven to be efficient when implemented at relevant temporal and spatial scales e.g. for *A*. *terrestris* in Franche-Comté, France^61–63^. This approach consists of synergistically manipulating the several factors that are known to directly or indirectly effect the spatial and temporal dynamics of the vole populations (e.g. mole control, habitat disturbance such as cattle trampling, short grass, plough rotation, targeted chemical control and trapping on early vole colonies, installation of buzzard roosts, vole-predator protection, etc.).

Managed grassland ecosystems all over Europe are suitable for *M*. *arvalis* and provide an extremely large range of landscape configurations and habitat productivities. However, comparisons are dependent on a clear description of context, hence on landscape- and habitat-description quality. This can be quite complex but essential in heterogeneous landscapes (Table 7), and scale dependencies must be taken into account^64^. This should lead to considering landscape characteristics on a large scale relevant to vole dispersal and predator populations (e.g. ≃100 km^−2^). A relatively large number of studies deal with vole cyclicity patterns, but few of them have monitored populations parameters (reproduction, mortality, etc.) associated with density variation in the long term. We believe that grassland vole species with a similar ecology can benefit from such studies and also provide relevant comparisons where their range offers a variety of landscapes. Based on our field experience, this is for instance the case for *A*. *terrestris*, and maybe *M*. *agrestis* in Europe (in places where *M*. *arvalis* is absent for the latter), *M*. *obscurus*, *M*. *gregalis*, *Ellobius tancrei*, *Lasiopodomys brandtii* in central Asia, *M*. *limnophilus*, *Phaiomys leucurus/Lasiopodomys fuscus* on the Tibetan plateau and its margins, all species extremely common with evidence of population outbreaks on large areas among a diversity of land uses and landscapes^65–67^.

**Table 7:** Habitat variables of importance to describe landscape context for grassland voles. %, percentage of land cover.

Habitat type	Subtype	Variables	Examples
Optimal	Permanent grassland	%, productivity, grazing frequency, grass height, fragmentation	Pastures, meadows
	Semi-permanent grassland marginal grassy areas non-tilled/direct seeding crops	%	Alfa-alfa, clover, other legumes, etc.
		%	Grassy field margins
		%	Crops with permanent grass cover
Seasonal		%	Any crop tilled every year (e.g. cereals, rape, etc.)
Suboptimal		%	Forest, marshes, etc.

## Supplementary information

kml file of the study area
